# Targeting the ROR1 and ROR2 receptors in epithelial ovarian cancer inhibits cell migration and invasion

**DOI:** 10.18632/oncotarget.5643

**Published:** 2015-10-20

**Authors:** Claire Henry, Estelle Llamosas, Alexandra Knipprath-Mészáros, Andreas Schoetzau, Ellen Obermann, Maya Fuenfschilling, Rosemarie Caduff, Daniel Fink, Neville Hacker, Robyn Ward, Viola Heinzelmann-Schwarz, Caroline Ford

**Affiliations:** ^1^ Metastasis Research Group, Adult Cancer Program, Lowy Cancer Research Centre and Prince of Wales Clinical School, Faculty of Medicine, University of New South Wales, New South Wales, Australia; ^2^ Department of Gynecology and Gynecological Oncology, Hospital for Women, University Hospital Basel, University of Basel, Basel, Switzerland; ^3^ Department of Pathology, University Hospital Basel, University of Basel, Basel, Switzerland; ^4^ Department of Pathology, University Hospital Zurich, Zurich, Switzerland; ^5^ Department of Gynecology, University Hospital Zurich, Zurich, Switzerland; ^6^ Gynaecological Cancer Centre, Royal Hospital for Women, Sydney, Australia; ^7^ Department of Research, The University of Queensland, Brisbane, Queensland, Australia

**Keywords:** ROR2, ROR1, Wnt signalling, epithelial ovarian cancer, metastasis

## Abstract

**AIM:**

In recent years, the Wnt signalling pathway has been implicated in epithelial ovarian cancer and its members have potential as diagnostic, prognostic and therapeutic targets. Here we investigated the role of two Wnt receptor tyrosine kinases (RTKs), ROR1 and ROR2, and their putative ligand, Wnt5a, in ovarian cancer.

**METHODS:**

Immunohistochemistry for ROR2 was performed in a large patient cohort, including benign controls, borderline tumours and epithelial ovarian cancer. In addition, siRNA was used to silence ROR1, ROR2 and Wnt5a individually, and together, in two ovarian cancer cell lines, and the effects on cell proliferation, adhesion, migration and invasion were measured.

**RESULTS:**

ROR2 expression is significantly increased in ovarian cancer patients compared to patients with benign disease. *In vitro* assays showed that silencing either receptor inhibits ovarian cancer cell migration and invasion, and concurrently silencing both receptors has an even stronger inhibitory effect on proliferation, migration and invasion.

**CONCLUSIONS:**

ROR2 expression is increased in epithelial ovarian cancer, and silencing ROR2 and its sister receptor ROR1 has a strong inhibitory effect on the ability of ovarian cancer cells to proliferate, migrate and invade through an extracellular matrix.

## INTRODUCTION

In stark contrast to other cancers, such as breast, the survival rate for ovarian cancer has not changed significantly in the last thirty years. Challenges remain in understanding the aetiology of the disease, how to detect it early enough for treatment to be effective, and how to stop the disease metastasising throughout the body. Though research has shown that there are diverse molecular subtypes of ovarian cancer, this genetic information has largely not yet been translated into changes in clinical practice [1–3]. Further research into the molecular changes underpinning the disease needs to be conducted to identify those key pathways responsible for cancer initiation and progression. Furthermore, once pathways are identified, specific biomarkers need to be pursued and robustly investigated to determine their potential as drug targets.

We, and others have shown that the Wnt signalling pathway is one such pathway involved in ovarian carcinogenesis, metastasis and drug resistance [4–8]. We recently reported that the Wnt ligand, Wnt5a, is upregulated in ovarian cancer patients and modelling this *in vitro* leads to increased cell proliferation and migration [5]. Wnt5a is known to bind to and signal through Frizzled receptors to initiate β-catenin independent Wnt signalling, but has also been shown to act as a ligand for the previously named “orphan” receptor, ROR2. ROR2 is a member of the receptor tyrosine kinase superfamily and its overexpression has been reported in many human cancers over the last few years [9–15], though little has been reported as to the downstream signalling cascade.

ROR1, the sister receptor of ROR2, has recently emerged as a critical modulator of Epithelial-Mesenchymal Transition (EMT) in breast cancer [16, 17]. Recent studies have reported a correlation between ROR1 expression and poor clinical outcome including relapse and survival in ovarian cancer patients [18, 19] and have even linked ROR1 to ovarian cancer stem cell migration and growth of tumour xenografts [18, 19].

Based on our previous results supporting the upregulation of β-catenin independent Wnt signalling in ovarian cancer [5, 20], we hypothesised that ROR2 would also be upregulated in ovarian cancer patients.

In addition, we also sought to determine the therapeutic potential of targeting these receptors by performing an extensive suite of *in vitro* experiments, exploring the functional role of ROR2, its sister receptor, ROR1 and putative ligand, Wnt5a in ovarian cancer.

These studies have confirmed the importance of ROR1 and ROR2 in the Wnt signalling pathway, and provided a strong argument for these receptors potential as clinical targets.

## RESULTS

### Expression of ROR2 is increased in epithelial ovarian cancer patients compared to benign controls

**Figure 1:**
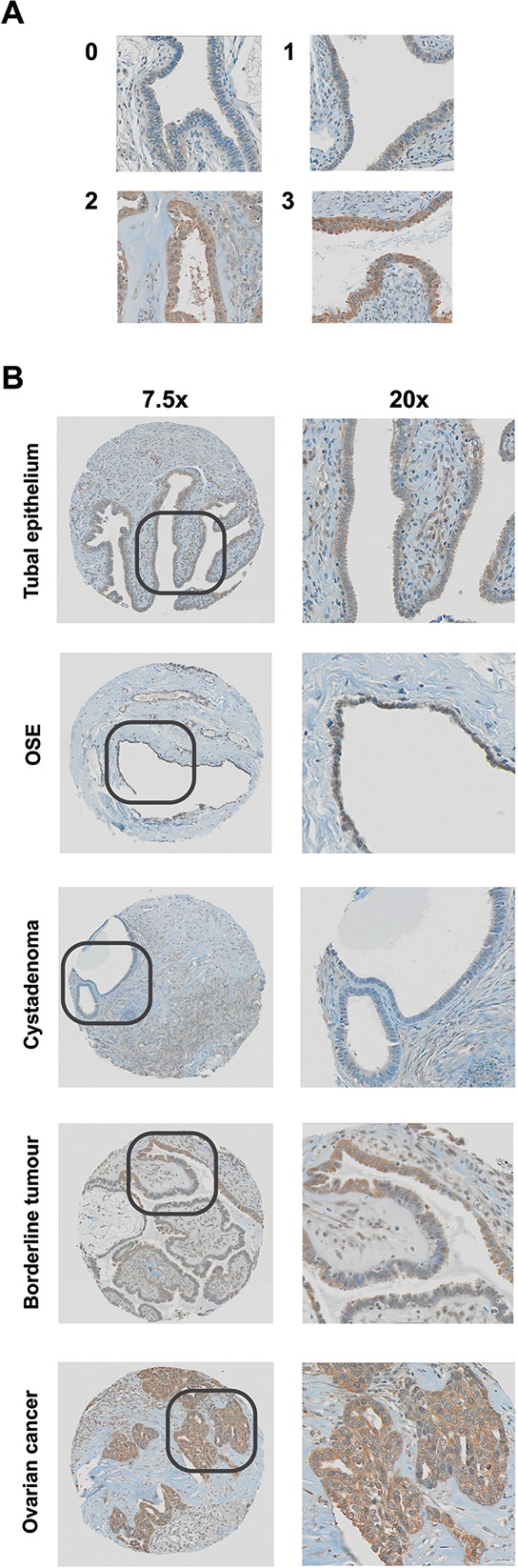
ROR2 protein expression as measured by immunohistochemistry **A.** Representative staining at 0, 1, 2 and 3 intensity. **B.** Representative IHC staining in tubal epithelium, ovarian surface epithelium (OSE), cystadenoma, borderline, and ovarian cancer samples.

**Figure 2:**
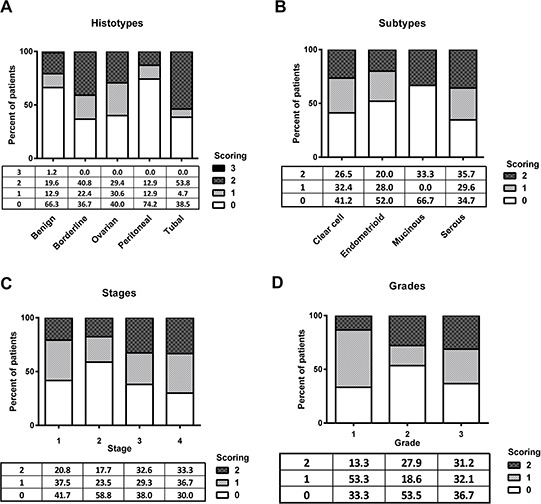
ROR2 expression is elevated in epithelial ovarian cancer **A.** Expression of ROR2 in benign, borderline tumours, ovarian cancer, peritoneal cancer and tubal cancer patients, expressed as a percentage of total. **B.** ROR2 expression in ovarian cancer patients stratified by subtype. **C.** ROR2 expression in ovarian cancer patients stratified by stage. **D.** ROR2 expression in ovarian cancer patients stratified by grade.

Tissue sections from ovarian cancer patients had a significantly higher expression of ROR2 than tissue sections taken from benign controls (Figure 1, Figure 2A, *p* = 0.0017). ROR2 expression was also elevated in tissue sections from patients with borderline tumours compared to benign controls (Figure 2A, *p* = 0.017). There was no significant difference observed between ROR2 expression in borderline tumours and ovarian cancer patients.

### ROR2 expression association with clinicopathological parameters

No differences in expression of ROR2 were observed between the four main subtypes of epithelial ovarian cancer: serous, endometrioid, clear cell and mucinous (Figure 2B). There was no association between ROR2 expression and stage (Figure 2C), yet a trend was observed between ROR2 expression and cancer grade. Patients with higher grade tumours were more likely to exhibit high ROR2 expression (Figure 2D, *p* = 0.08). Individual scores for each parameter are shown in Table 1. Seven patients were missing information and 3 patients were missing information on grade, and were therefore excluded from further analysis.

**Table 1:** Patient cohort characteristics

	Patient samples	Staining intensity				Stage				Grade		
		0	1	2	3	1	2	3	4	1	2	3
**ALL**		426	222	89	113	2						
**BENIGN**												
**Healthy controls**	163	108	21	32	2							
**INVASIVE / NON INVASIVE TUMORS**												
**Borderline tumors**	49	18	11	20	0							
**Cancers**												
**Ovarian cancers**	170	68	52	50	0							
Serous	98	34	29	35	0	9	9	55	20	3	23	69
Endometrioid	25	13	7	5	0	9	4	12	0	7	12	6
Clear cell	34	14	11	9	0	2	4	20	6	0	6	28
Transitional cell	8	4	4	0	0	2	0	2	4	4	0	4
Mucinous	3	2	0	1	0	2	0	1	0	1	2	0
Other	2	1	1	0	0	0	0	2	0	0	0	2
**Peritoneal**	31	23	4	4	0							
**Tubal**	13	5	1	7	0							

### Expression of ROR2 in epithelial ovarian cancer patients and patient survival

From the whole cohort, 120 epithelial ovarian cancer patients showed a recurrence of their disease and were therefore included in the relapse-free survival analysis. No association was observed between ROR2 expression at time of surgery and relapse-free survival in this patient cohort (Supplementary Figure S1A). Similarly, no difference in overall survival was observed within the critical period of the first few years following diagnosis when patients were stratified according to their ROR2 expression status at surgery. We did observe in our cohort over a very long follow-up period of 20 years that those patients with good outcomes (defined as surviving past 5 years diagnosis), lacking ROR2 expression (0) had a worse prognosis than those expressing ROR2 (1, 2) (Supplementary Figure S1B).

### ROR2 regulates ovarian cancer cell migration and invasion

**Figure 3:**
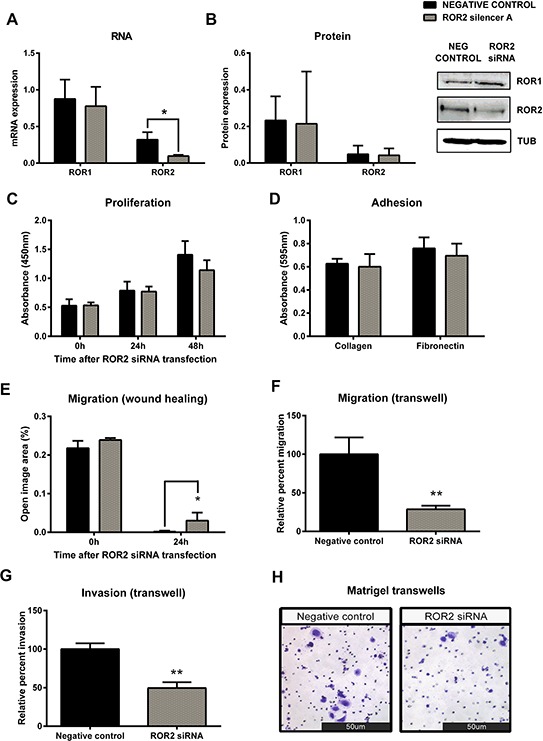
Knockdown of ROR2 in serous ovarian cancer cells inhibits cell migration **A.** ROR2 is decreased at the mRNA level following siRNA (A) induced knockdown in serous ovarian cancer (OVCAR3) cells. No effect on ROR1 mRNA level. qRT-PCR was performed in triplicate and normalised to three different housekeeping genes (SDHA, HSPCB, RPL13A). Results represent an average of four experiments. Error bars represent the s.d of the mean. **P < 0.01. **B.** Densitometric analysis of ROR1 and ROR2 protein levels from three separate experiments. Representative immunoblots showing ROR2 knockdown at the protein level in OVCAR3 cells. No effect on ROR1 protein level. Top panel: ROR1, middle panel: ROR2, bottom panel: α-tubulin. **C.** Cell proliferation is slightly decreased following ROR2 knockdown in OVCAR3 cells over a 48–72 hours period, but not significantly. Results represent the average of three independent experiments. Error bars represent the s.d of the mean. **D.** ROR2 knockdown has no effect on the adhesion of OVCAR3 cells to collagen or fibronectin. Results represent the average of 3 experiments. **E.** Cell migration performed using the wound healing assay is significantly decreased following ROR2 knockdown in OVCAR3 cells. Results represent an average of three experiments. Error bars represent the s.d of the mean. *P < 0.05. **F.** Relative cell migration performed using the transwell migration assay is significantly decreased following ROR2 knockdown in OVCAR3 cells. Results represent an average of three experiments. Error bars represent the s.d of the mean. **P < 0.01. **G.** Relative cell invasion performed using the matrigel pre coated transwell assay significantly decreased following ROR2 knockdown in OVCAR3 cells. Results represent the average of three experiments. Error bars represent the s.d of the mean. **P < 0.01. **H.** Representative picture of OVCAR3 cells invading matrigel over 48 hours.

The serous ovarian cancer cell line OVCAR3 was chosen for ROR2 knockdown assays due to its expression of both ROR1 and ROR2 at the mRNA and protein level. Successful transfection of ROR2 siRNA significantly decreased mRNA (Figure 3A, *p* < 0.05) and protein (Figure 3B) levels of ROR2, and had no effect on the level of ROR1, as expected. ROR2 knockdown in OVCAR3 slightly decreased proliferation however this did not reach significance (Figure 3C). ROR2 knockdown had no effect on cell adhesion to collagen or fibronectin (Figure 3D). ROR2 knockdown in OVCAR3 significantly decreased cell migration in the two-dimensional (2D) wound healing assay (Figure 3E, *p* < 0.05). Control cells migrated to completely close the wound within 24 hours, whereas ROR2 knockdown cells were unable to close the wound, leaving visible space (quantitated as “open image area”). To validate the findings from the wound healing assay and investigate the role of ROR2 in migration further, a three-dimensional (3D) transwell model was used, which measured the ability of the cells to migrate through pores to a chemoattractant. ROR2 knockdown significantly inhibited the ability of OVCAR3 to migrate through the transwell (Figure 3F, *p* < 0.01). To continue the investigation into cell invasion, a similar approach was used with pre-coated transwells, measuring the ability of the cells to invade through a matrigel layer before migrating through pores to a chemoattractant. ROR2 knockdown in OVCAR3 decreased invasion significantly (Figure 3G–3H, *p* < 0.01).

To validate these results, a second siRNA targeting ROR2, ROR2 siRNA B was transfected into OVCAR3 cells and was also shown to inhibit cell migration and invasion (Supplementary Figure S2).

To further investigate the role of ROR2 in ovarian cancer, a third ROR2 knockdown was undertaken in the endometrioid ovarian cancer cell line, TOV112D. As well as possessing a mutation in β-catenin, this cell line has high levels of ROR2 expression and no detectable ROR1 expression (Supplementary Figure S7A). ROR2 knockdown in this cell line also significantly inhibited cell migration and seemed to inhibit invasion (Supplementary Figure S3).

### ROR1 regulates ovarian cancer migration and invasion

**Figure 4:**
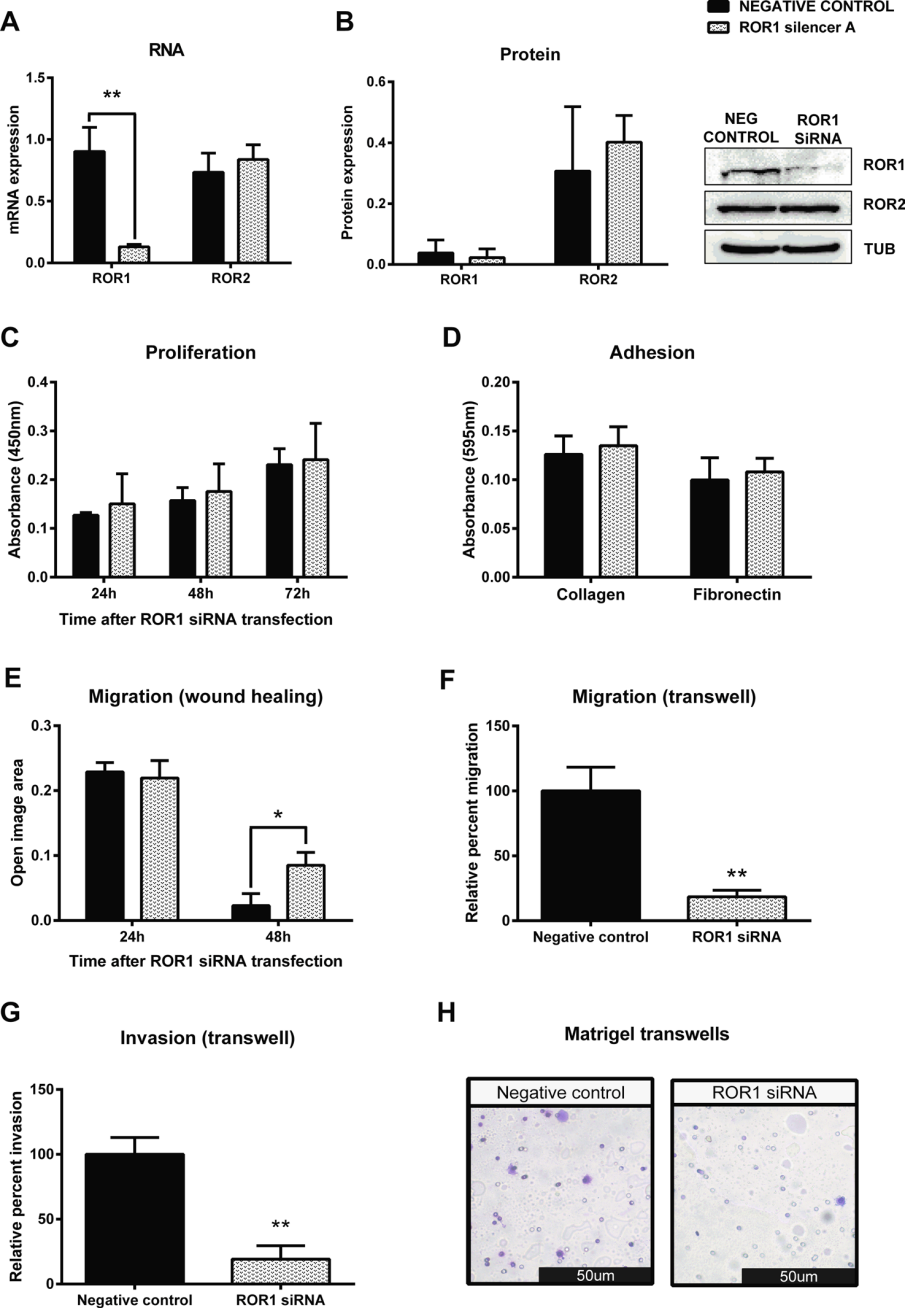
Knockdown of ROR1 in serous ovarian cancer cells decreases migration **A.** ROR1 is decreased at the mRNA level following ROR1 siRNA (A) induced knockdown in serous ovarian cancer (OVCAR3) cells. No effect on ROR2 mRNA level. qRT-PCR was performed in triplicate and normalised to three different housekeeping genes (SDHA, HSPCB, RPL13A). Results represent an average of three experiments. Error bars represent the s.d of the mean. **P < 0.01. **B.** Densitometric analysis of ROR1 and ROR2 protein levels from three separate experiments. Representative immunoblots showing ROR1 knockdown at the protein level in OVCAR3 cells. No effect on ROR2 protein level. Top panel: ROR1, middle panel: ROR2, bottom panel: α-tubulin. **C.** Cell proliferation does not change following ROR1 knockdown in OVCAR3 cells over a 48–72 hour period. Results represent the average of three independent experiments. Error bars represent the s.d of the mean. **D.** ROR1 knockdown has no effect on the adhesion of OVCAR3 cells to collagen or fibronectin. Results represent the average of 3 experiments and error bars represent the s.d of the mean. **E.** Cell migration performed using the wound healing assay decreases following ROR1 knockdown in OVCAR3 cells. Results represent an average of three experiments. Error bars represent the s.d of the mean. *P < 0.05. **F.** Relative cell migration performed using the transwell migration assay is significantly decreased following ROR1 knockdown in OVCAR3 cells. Results represent an average of three experiments. Error bars represent the s.d of the mean. **P < 0.01. **G.** Relative cell invasion performed using the matrigel pre coated transwell assay is significantly decreased following ROR1 knockdown in OVCAR3 cells. Results represent the average of three experiments. Error bars represent the s.d of the mean. **P < 0.01. **H.** Representative picture of OVCAR3 cells invading matrigel over 48 hours.

Successful transfection of ROR1 siRNA significantly decreased mRNA (Figure 4A, *p* < 0.01) and protein (Figure 4B) levels of ROR1, and had no effect on the level of ROR2 (Figure 4A–4B) in the OVCAR3 cell line. ROR1 knockdown had no effect on cell proliferation (Figure 4C), nor on cell adhesion to collagen or fibronectin (Figure 4D). However, ROR1 knockdown in OVCAR3 significantly decreased cell migration in both the 2D wound healing assay (Figure 4E, *p* < 0.05) and the 3D transwell assay (Figure 4F, *p* < 0.01). In addition, ROR1 knockdown significantly inhibited the invasive ability of OVCAR3 cells (Figure 4G–4H, *p* < 0.01).

To validate these results and rule off any off-target effects, a second siRNA targeting ROR1, ROR1 siRNA B, was transfected into OVCAR3 cells and also shown to inhibit cell migration and invasion (Supplementary Figure S4).

It is important to note that our model “normal” epithelial ovarian cell line, HOSE6.3, had high expression of ROR1 at both the mRNA and protein level (Supplementary Figure S5, Supplementary Figure S7A). Knocking down ROR1 in these cells (Supplementary Figure S5A–5B), had no effect on cell proliferation, adhesion or migration (Supplementary Figure S5C–S5E).

### ROR1 and ROR2 synergistically regulate ovarian cancer migration and invasion

**Figure 5:**
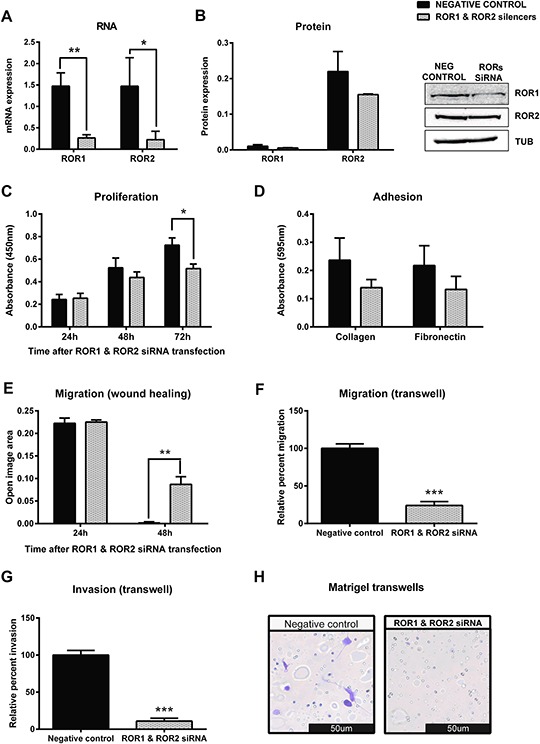
Simultaneous knockdown of ROR1 and ROR2 in serous ovarian cancer cells decreases migration and proliferation **A.** ROR1 and ROR2 are decreased at the mRNA level following siRNA (A) induced knockdown in serous ovarian cancer (OVCAR3) cells. qRT-PCR was performed in triplicate and normalised to three different housekeeping genes (SDHA, HSPCB, RPL13A). Results represent an average of three experiments. Error bars represent the s.d of the mean. **P < 0.01, *P < 0.05. **B.** Densitometric analysis of ROR1 and ROR2 protein levels from three separate experiments. Representative immunoblots showing ROR1 and ROR2 knockdown at the protein level in OVCAR3 cells. Top panel: ROR1, middle panel: ROR2, bottom panel: α-tubulin. **C.** Cell proliferation decreases following ROR1 and ROR2 knockdown in OVCAR3 cells over a 48–72 hour period. Results represent the average of three independent experiments. Error bars represent the s.d of the mean. *P < 0.05. **D.** ROR1 and ROR2 knockdown may decrease the adhesion of OVCAR3 cells to collagen or fibronectin, but not significantly. Collagen *p* = 0.115, Fibronectin *p* = 0.155. Results represent the average of 3 experiments and error bars represent the s.d of the mean. **E.** Cell migration performed using the wound healing assay decreases following ROR1 and ROR2 knockdown in OVCAR3 cells. Results represent an average of three experiments. Error bars represent the s.d of the mean. **P < 0.01. **F.** Relative cell migration performed using the transwell migration assay is significantly decreased following ROR1 and ROR2 knockdown in OVCAR3 cells. Results represent an average of three experiments. Error bars represent the s.d of the mean. ***P < 0.001. **G.** Relative cell invasion performed using the matrigel pre coated transwell assay is significantly decreased following ROR1 and ROR2 knockdown in OVCAR3 cells. Results represent the average of three experiments. Error bars represent the s.d of the mean. ***P < 0.001. **H.** Representative picture of OVCAR3 cells invading matrigel over 48 hours.

As the OVCAR3 cell line expressed both ROR1 and ROR2, we were interested to investigate the synergistic effect of ROR1 and ROR2 on cancer cell behaviour. Successful co-transfection of ROR1 and ROR2 siRNA significantly decreased mRNA (Figure 5A, *p* < 0.01) and protein (Figure 5B) levels of ROR1 and ROR2. Double knockdown of ROR1 and ROR2 significantly decreased cell proliferation 72 hours after transfection (Figure 5C *p* < 0.05), and reduced adhesion to collagen and fibronectin, though this did not reach significance (Figure 5D). Most interestingly, ROR1 and ROR2 knockdown in OVCAR3 significantly decreased cell migration in the 2D wound healing assay (Figure 5E, *p* < 0.001) and 3D transwell migration assays (Figure 5F, *p* < 0.001). This inhibition was stronger than that seen with ROR1 or ROR2 knockdown alone. Similarly, this synergistic effect was also observed in the transwell invasion (Figure 5G–5H, *p* < 0.001) assays, where the double ROR knockdown dramatically halted the ability of the OVCAR3 cells to invade through a layer of matrigel.

### Wnt5a regulates ovarian cancer migration and invasion

**Figure 6:**
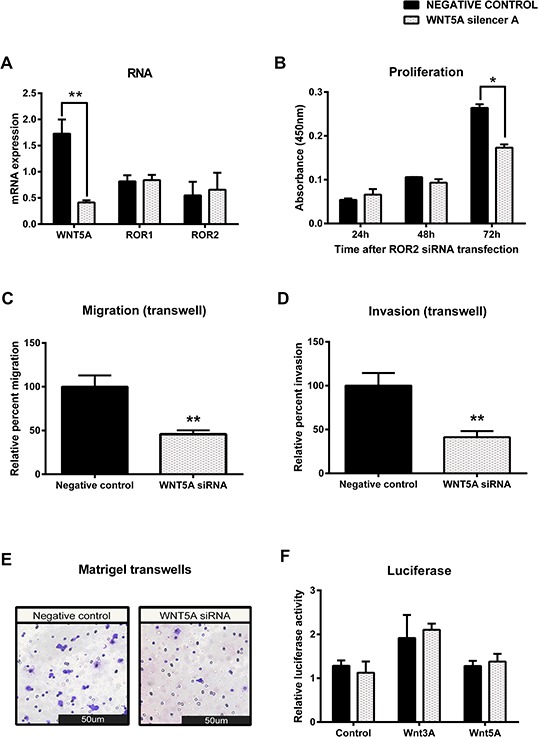
Knockdown of WNT5A in serous ovarian cancer decreases migration and invasion **A.** WNT5A is decreased at the mRNA level following siRNA (A) induced knockdown in serous ovarian cancer (OVCAR3) cells. No effect on ROR1 or ROR2 mRNA level. qRT-PCR was performed in triplicate and normalised to three different housekeeping genes (SDHA, HSPCB, RPL13A). Results represent an average of three experiments. Error bars represent the s.d of the mean. **P < 0.01. **B.** Cell proliferation decreases following WNT5A knockdown in OVCAR3 cells over a 48–72 hour period, however did not come to significance (*P* = 0.076). Results represent the average of three independent experiments. Error bars represent the s.d of the mean. **C.** Relative cell migration performed using the transwell migration assay is significantly decreased following WNT5A knockdown in OVCAR3 cells. Results represent an average of three experiments. Error bars represent the s.d of the mean. **P < 0.01. **D.** Relative cell invasion performed using the matrigel pre coated transwell assay is significantly decreased following WNT5A knockdown in OVCAR3 cells. Results represent the average of three experiments. Error bars represent the s.d of the mean. **P < 0.01. **E.** Representative picture of OVCAR3 cells invading matrigel over 48 hours. **F.** Luciferase assay determined no change in β-catenin dependent signalling after WNT5A knockdown in OVCAR3. Relative β-catenin driven transcription activity was calculated as a TOP/FOP ratio in triplicate wells. Results represent an average of three experiments. Error bars represent the s.d of the mean.

To investigate mechanisms of ROR signalling in the OVCAR3 cell line, the putative ROR ligand, Wnt5a was also silenced using siRNA. Successful transfection of Wnt5a siRNA significantly decreased mRNA (Figure 6A, *p* < 0.01) levels of Wnt5a, and significantly decreased cell proliferation 72 hours after transfection (Figure 6B *p* < 0.05). Migration and invasion of OVCAR3 was also significantly inhibited by Wnt5a knockdown (Figure 6C–6E *p* < 0.05). A luciferase assay was used to investigate TCF/LEF transcriptional activity and thus the activation of the β-catenin dependent pathway. Interestingly there was no change in activity between the control and Wnt5a knockdown cells (Figure 6F).

To validate these results and rule out any off-target effects, a second siRNA targeting Wnt5a, Wnt5a siRNA B was transfected into OVCAR3 cells and also shown to significantly inhibit cell migration and invasion (Supplementary Figure S6).

**Figure 7:**
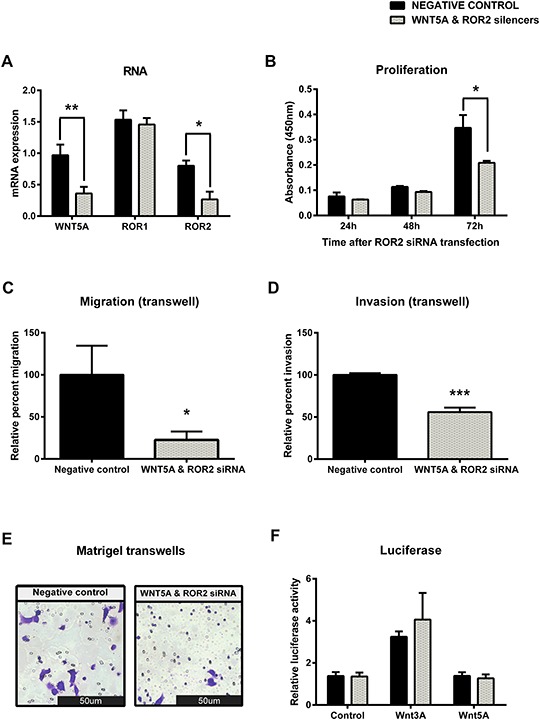
Simultaneous knockdown of WNT5A and ROR2 in serous ovarian cancer decreases proliferation, migration and invasion **A.** WNT5A and ROR2 are decreased at the mRNA level following siRNA (A) induced knockdown in serous ovarian cancer (OVCAR3) cells. No effect on ROR1 mRNA level. qRT-PCR was performed in triplicate and normalised to three different housekeeping genes (SDHA, HSPCB, RPL13A). Results represent an average of three experiments. Error bars represent the s.d of the mean. *P < 0.05, **P < 0.01. **B.** Cell proliferation decreases following WNT5A and ROR2 knockdown in OVCAR3 cells after 72 hours. Results represent the average of three independent experiments. Error bars represent the s.d of the mean. *P < 0.05. **C.** Relative cell migration performed using the transwell migration assay is significantly decreased following WNT5A and ROR2 knockdown in OVCAR3 cells. Results represent an average of three experiments. Error bars represent the s.d of the mean. *P < 0.05. **D.** Relative cell invasion performed using the matrigel pre coated transwell assay is significantly decreased following WNT5A and ROR2 knockdown in OVCAR3 cells. Results represent the average of three experiments. Error bars represent the s.d of the mean. ***P < 0.001. **E.** Representative picture of OVCAR3 cells invading matrigel over 48 hours. **F.** Luciferase assay determined a slight non-significant increase in WNT3A stimulated β-catenin dependent signalling after WNT5A and ROR2 knockdown in OVCAR3. Relative β-catenin driven transcription activity was calculated as a TOP/FOP ratio in triplicate wells. Results represent an average of three experiments. Error bars represent the s.d of the mean.

To further investigate Wnt5a induced β-catenin independent signalling through ROR2, a double knockdown of Wnt5a and ROR2 was performed on the OVCAR3 cells. Successful co transfection was seen at the transcriptional level (Figure 7A, *p* < 0.01 and *p* < 0.05) which resulted in a significant decrease in proliferation at 72 hours after transfection (Figure 7B, *p* < 0.05). Double ROR2 and Wnt5a silencing in OVCAR3 also resulted in significant inhibition of 3D migration and invasion (Figure 7C–7E, *p* < 0.05 and *p* < 0.001). A slight increase in Wnt3a induced β-catenin dependent signalling was observed in the double ROR2/Wnt5a silenced OVCAR3 cells, however interestingly again, we found that there was no significant change in β-catenin dependent pathway activation (Figure 7F).

### ROR1 and ROR2 may regulate separate signalling pathways

Both ROR1 and ROR2 have been linked to the β-catenin independent Wnt signalling pathway, but little is known about the downstream targets of ROR1 and ROR2 in cancer, and their effect on the β-catenin dependent Wnt signalling pathway. We therefore investigated a number of key Wnt and EMT related genes in our knockdown samples.

Knockdown of ROR2 in OVCAR3 cells resulted in a significant increase in the Wnt target, Axin2 (Supplementary Figure S7B, *p* < 0.01) and decrease in Jnk (Supplementary Figure S7A, *p* < 0.01), and significantly reduced levels of the EMT marker, Vimentin (Supplementary Figure S7B, *p* < 0.05).

Knockdown of ROR1 in OVCAR3 cells significantly increased Axin2 mRNA expression (Supplementary Figure S7C, *p* < 0.05) and had no effect on Jnk. No effect on EMT markers was observed.

Double knockdown of ROR1 and ROR2 in OVCAR3 cells appeared to increase both Axin2 and Jnk mRNA expression (Supplementary Figure S7D). The effect on EMT markers was mixed, with Twist mRNA expression levels significantly increased (Supplementary Figure S7D, *p* < 0.05), no effect on Vimentin, and P-cadherin levels significantly decreased (Supplementary Figure S7D, *p* < 0.05).

Knockdown of ROR2 in TOV112D cells resulted in a significant decrease in the Wnt targets, Axin2 (Supplementary Figure S7A, *p* < 0.05) and Jnk (Supplementary Figure S7A, *p* < 0.05), and significantly reduced levels of the EMT marker, Vimentin (Supplementary Figure S7A, *p* < 0.05).

The transcriptional levels of the Wnt gatekeeper, SFRP4, were also investigated. Knockdown of ROR1 or ROR2 alone has no effect on mRNA levels of SFRP4, but double knockdown of ROR1 and ROR2 significantly increased SFRP4 expression (Supplementary Figure S7D, *p* < 0.05).

## DISCUSSION

We have previously shown in a large patient cohort that Wnt5a is upregulated in epithelial ovarian cancer compared to healthy controls [5]. Here, we have shown that the receptor for the Wnt5a ligand, ROR2, is also increased in epithelial ovarian cancer patients (Figures 1, 2). No difference was observed between different subtypes of ovarian cancer, suggesting that ROR signalling is not subtype specific. This aligns with our previous results on two other important Wnt genes, SFRP4 and Wnt5a [5, 20].

ROR2 expression was not associated with relapse free survival or overall survival within the first few years following diagnosis and surgery. However, our long follow-up data (up to 20 years) did indicate that that patients lacking ROR2 expression had a shorter overall survival compared to patients with ROR2 expression. This trend is opposite to that reported of ROR1 in ovarian cancer [18], and ROR2 in other tumours, where ROR2 expression has been associated with worse overall or disease specific survival [10, 13, 21]. These studies all had significantly shorter follow-up times. Therefore this difference may be due to the multiple confounders that cannot be controlled for in long-term survival data, or may highlight a different function for ROR2 in ovarian cancer. It is also important to note that ROR2 IHC is performed on samples taken at the time of surgery, and our samples have not been stratified into those patients that presented with widespread or localised disease. We are now investigating ROR2 expression in other ovarian cancer cohorts to see if this survival association holds true. If confirmed, it may highlight that ROR2 influences migration and invasion at a particular time point in oncogenesis, which could be more associated with metastasis than primary tumour formation

Recently, two papers have revealed that ROR1 is overexpressed in ovarian cancer and can be targeted for anti-cancer stem cell therapy *in vivo*. In a cohort of 100 ovarian cancer patients, ROR1 was shown to be increased and statistically correlated with FIGO stage, tumour grade and lymph node metastasis [18]. Additionally, patients with high ROR1 expression had poor disease free and overall survival. A second recent paper confirmed the relationship of ROR1 to poor survival in the publically available database Gene Expression Omnibus, and also found that ovarian cancer cells with high ROR1 expression also featured stem cell like gene signatures [19]. The use of primary cancer cells and investigations into ovarian cancer spheroids and tumourigenesis *in vivo* supports the role of ROR1 in ovarian cancer. Furthermore, the authors linked ROR1 with EMT, and showed that their anti-ROR1 monoclonal antibody inhibited ovarian cancer xenograft growth and reduced expression of EMT markers [19].

Our study confirms the role of ROR1 in ovarian cancer progression, via our transwell migration and invasion assays (Figure 4E–4H and Figure 5E–5H). Moreover for the first time our study also includes ROR2, and shows that it is also overexpressed in ovarian cancer, and may play a role in cancer progression. A very recent paper reported that ROR2 is over-expressed in cervical cancer and associated with unfavourable prognosis and tumour progression [13]. Thus, the combination of our results here in ovarian cancer and recent others [13, 16, 19], suggests that in gynaecological cancers both ROR1 and ROR2 may be over expressed, and important for disease progression. It is imperative that the relationship of the two receptors is investigated, to further elucidate their individual and combined roles in the progression and metastasis of disease.

Modulation of ROR1 and ROR2 *in vitro* highlighted distinct roles for each receptor, as well as some synergistic effects. It is important to note that though receptor levels were significantly reduced, neither receptor was silenced completely, and as expected, the efficacy of silencing seemed to determine the degree of migration and invasion inhibition. Thus, further studies should include stable knockdown cell lines through short hairpin RNA (shRNA).

Knockdown of ROR1 had no effect on proliferation (Figure 4C, Supplementary Figure S4C), while knockdown of ROR2 appeared to decrease proliferation, though this was not statistically significant (Figure 3C, Supplementary Figure S3C). When both receptors were knocked down, proliferation was significantly decreased (Figure 5C, *p* < 0.05). These results suggest that ROR2 expression may be more important than ROR1 for ovarian cancer cell proliferation, but that targeting them together may be effective in inhibiting proliferation. To further confirm this, Wnt5a ligand knockdown also inhibited proliferation (Figure 6B) which suggests that Wnt5a mediated signalling through both ROR1 and ROR2 may be essential for cell proliferation.

Knockdown of either ROR1 or ROR2 alone had no effect on cell adhesion to collagen or fibronectin (Figure 3D, Figure 4D). Knockdown of both ROR1 and ROR2 reduced adhesion to both collagen and fibronectin, but this did not reach statistical significance (Figure 5D), suggesting that the ROR receptors do not play a major role in regulating ovarian cancer cell adhesion.

The strongest effects of ROR knockdown were seen in the migration and invasion assays. Knocking down either receptor significantly inhibited cell migration in both 2D and 3D assays (Figure 3E–3F, Figure 4E–4F), and knocking down both receptors strengthened this inhibition further (Figure 5E–5F, *p* < 0.001). ROR1 knockdown significantly inhibited cell invasion (Figure 4G, *p* < 0.01), as did ROR2 knockdown (Figure 3G, *p* < 0.05). However, knocking down both ROR1 and ROR2, further inhibited cell invasion (Figure 5G, *p* < 0.001). These results support the role of ROR1 and ROR2 in governing ovarian cancer cell migration and invasion, and suggest that blocking these receptors may inhibit cancer progression.

Wnt5a has been demonstrated to bind to both ROR1 and ROR2, so we also investigated the effect of silencing this β-catenin independent ligand in our cell line models. Knockdown of Wnt5a inhibited cell proliferation, migration and invasion (Figure 6C–6D, Supplementary Figure S6), and this effect was enhanced when ROR2 was silenced at the same time (Figure 7).

The exact mode of signalling of ROR1 and ROR2 has yet to be elucidated, yet our functional results suggest that they may be acting in separate pathways to provide different outcomes on migration and invasion. Therefore we investigated a number of downstream genes involved in β-catenin dependent, β-catenin independent and EMT signalling (Supplementary Figure S7). It was interesting to see that AXIN2, a β-catenin dependent target was increased in either ROR1 or ROR2 knockdown, however when both receptors where knockdown together, this increase became non-significant. JNK, a β-catenin independent target, decreased upon ROR2 knockdown however did not change in either ROR1 or double knockdown, indicating that ROR2 may only signal through the PCP pathway, whereas ROR1 may not. This can be supported by other studies, where it was shown that ROR2 binds to Wnt5a to activate the small GTPases RhoA and Rac, and subsequently initiates the Jun Terminal Kinase (JNK) pathway by Actin binding protein filamin A to regulate cell polarity and migration [22, 23]. Additionally, the interaction of ROR2 with Frizzled7 is important in Wnt5a induced AP-1 transcription factor activation, downstream of Rac1 and JNK [24]. AP-1 is an important transcription factor that is able to regulate the expression of many genes involved in cellular proliferation and survival. It may be through this arm of signalling that ROR2 can regulate cell proliferation as seen in our ovarian cancer cell line models.

The EMT gene TWIST increased upon ROR2 and double ROR1/ROR2 knockdown however ROR1 alone had no effect. Additionally, the EMT gene Vimentin decreased upon ROR2 and ROR1 single knockdowns, but seemed to slightly increase in the double knockdown (non-significantly). This may indicate that ROR2 feeds more into TWIST regulation than ROR1, and single knockdowns may allow for balance in signalling through the alternative ROR receptor, however when both receptors are knocked down, regulation is lost.

ROR1 may be involved in this regulation of signalling. ROR1 has been shown to also bind to Wnt5a, but to activate NF-kB [25], a family of transcription factors largely involved in inflammation and identified as the important link between the inflammatory microenvironment and progression to malignancy [26]. Interestingly, ROR1 has also been shown to be a docking site to mediate dependent c-Src and EGFR activation, leading to downstream targets of PI3K, AKT and NFAT signalling [27]. All of these subsequent pathway activations lead to cellular changes in proliferation, differentiation, migration, survival and metabolism. Interestingly, ROR1 has also been shown to translocate to the nucleus and act as a transcription factor which may explain its role in pathway regulation [28, 29]. However, these unique characteristics of ROR1 have not been reported in ovarian cancer, and may be specific in each cell type, as has been reported for ROR1 and EGFR association in lung cancer models [27].

ROR1 and ROR2 may also signal through different co-receptors or ligands. ROR2 was shown to signal through FZD7 to regulate cell migration through the β-catenin independent pathway in mouse fibroblast cells [24], but was also shown to signal through FZD2 in association with LRP5/6 in lung epithelial cells to modulate β-catenin dependent signalling. At present, ROR1 has only been shown to signal through binding with Wnt5a [25]. Our luciferase assay results (Figures 6F and 7F) interestingly do not reveal any change in β-catenin dependent signalling after Wnt5a or double Wnt5a and ROR2 knockdown. This indicates to us that there is a complex signalling network in play and that the ROR receptors may not feed into one single arm, yet influence a number of surrounding networks as discussed. It will be important to elucidate the mode in which ROR1 and ROR2 signal in epithelial ovarian cancer.

We have shown for the first time that both ROR1 and ROR2 regulate ovarian cancer cell migration and invasion. However, transwell membranes clearly do not mimic the complex inflammatory tumour micro environment in which ovarian cancer grows. Therefore it would be essential to continue further research into 3D culture methods and mouse models, to further validate the feasibility of therapeutically targeting these receptors. Our supplementary results show (Supplementary Figure S5) that targeting these receptors may not have effects on normal cells, as our ROR1 siRNA transfected HOSE6.3 normal cell line resulted in no changes to cellular proliferation, adhesion or migration. This indicates an exciting area for drug development, as minimal patient side effects may be seen, and, for the first time, a monoclonal ROR1 targeting antibody did not observe any off-target toxicity in preclinical studies and is now available in a phase I trial for Chronic Lymphocytic Leukemia [30]. Furthermore transcriptionally, no Wnt downstream targets were altered (Supplementary Figure S7F) in our HOSE6.3 cells. An explanation for this may be that the ROR1 receptor in normal cell lines may be present in the immature non-glycosylated form, and in the mature, active form in our ovarian cancer cell lines, as seen in a recent study [31].

The molecular changes underpinning ovarian cancer and in particular, ovarian cancer progression and metastasis, remain understudied. Important large scale sequencing projects are beginning to reveal the heterogeneity of this disease and confirm the importance of signalling pathways, including Wnt signalling. As cell surface receptor tyrosine kinases, ROR1 and ROR2 may play a key role in regulating this pathway and warrant further investigation.

## MATERIALS AND METHODS

### Patient cohort

Ethics approval was obtained from the Swiss Ethical Cantonal Department SPUK (approval #StV06/2006) to collect samples from patients at the University Hospital Zurich, Switzerland. The total cohort of 426 samples included 163 benign, 49 ovarian borderline, 31 peritoneal cancers, 13 tubal cancers, and 170 epithelial ovarian cancer samples. Clinicopathological data was available for this cohort, including survival data. This patient cohort and the production of the tissue microarray have previously been described in detail [5, 20].

### ROR2 immunohistochemistry

A previously published Tissue Microarray was used for the immunohistochemistry (IHC) experiments [5, 20]. Briefly, 4 μm paraffin sections were deparaffinized. Antigen recovery was conducted using CC1 conditioning solution (pH 8.0) for 16 minutes. Staining was performed on a VENTANA BenchMark XT automated slide-processing system (Ventana Medical Systems, Tucson, AZ). Sections were then incubated with anti-ROR2 antibody (Sigma HPA021868) for 32 minutes. OptiView DAB IHC Detection Kit was used according to the manufacturer's recommendations (Ventana Medical Systems, Tucson, AZ). Tissue slides were counterstained with Hematoxylin. Sections were dehydrated and coverslipped with PERTEX (Histolab, Goteborg, Sweden).

Immunostaining was scored by the percentage and intensity of cells staining (0–100% of cells stained within one core; intensity 0–3+). Scoring was assessed by a gynecologist with experience in immunohistochemistry (A.K.), and a subset of cases was analysed by two pathologists (F.M, E.O). Discrepancies were resolved by consensus. ROR2 expression from all cores were averaged per patient.

### Cell culture

The ovarian cancer cell lines TOV112D (endometrioid subtype) and OVCAR3 (serous subtype), and transformed immortalised human ovarian surface epithelial cells (HOSE6.3) were obtained from the American Type Culture Collection (Manassas, VA, USA) and cultured as per ATCC recommendations. Briefly, HOSE6.3 was cultured in a 50:50 ratio of 199 (Sigma): MCDB105 media (Sigma) containing 10% fetal calf serum. The OVCAR3 and TOV112D ovarian cancer cells were cultured in RPMI and DMEM media respectively, both with 10% Fetal Bovine Serum. Media was supplemented with penicillin/streptomycin and GlutaMAX (Life Technologies). All cells were grown in a humidified atmosphere of 5% CO_2_, at 37°C and were repeatedly demonstrated to be free of mycoplasma contamination.

Transfections were conducted using Lipofectamine2000 according to manufacturer's specifications (Invitrogen, Carlsbad, CA, USA). In all transfection experiments, unless otherwise specified, 1 × 10^6^ cells were seeded into 6 well plates and serum starved overnight. Cells were transfected the following day, 24 hours after initial seeding. ROR silencing was achieved by transfecting cells with 90 pmol of either ROR1 (named ROR1 siRNA A, Ambion, Carlsbad, CA, USA #4390824, s9755) or ROR2 (named ROR2 siRNA A, Ambion, Carlsbad, CA, USA #4390824 s9759) targeting siRNA, or negative control scrambled siRNA. Wnt5a silencing was achieved by transfecting cells with 90pmol of Wnt5a siRNA (Ambion, Carlsbad, CA, USA, #4392420, s14972, named Wnt5a siRNA A). To rule out off-target effects, additional ROR1, ROR2 and Wnt5a silencers were also transfected (Ambion, Carlsbad, CA, USA ROR1 s9756 named ROR1 siRNA B, ROR2 s9758 named ROR2 siRNA B, ROR2 s9760 named ROR2 siRNA C, Wnt5a s14871 named Wnt5a siRNA B, Wnt5a s14873 named Wnt5a siRNA C). The transfection mixture was removed after 6–8 hours, washed with serum free media and replaced with complete media containing 10% FBS. Cells were allowed to grow for a further 48 hours and 72 hours, and then harvested for RNA and protein extraction respectively. The efficiency of ROR and Wnt5a knockdown was confirmed by Western blotting and quantitative real time polymerase chain reaction (qRT-PCR).

### Quantitative real-time reverse transcriptase PCR (qRT-PCR)

RNA extraction, cDNA synthesis and qRT-PCR were performed as described previously [7]. Samples were run in triplicate and normalised against the housekeeping genes succinate dehydrogenase complex subunit A (SDHA), 90 kDa heat shock protein 1 beta (HSPCB) and 60S ribosomal protein L13a (RPL13A) [32]. All experiments contained a negative cDNA control (non-reverse transcriptase reaction) for each sample as well as a negative water control. Expression of genes of interest was normalised against the geometric mean of the three housekeeping genes using the Vandesompele normalisation method [33]. Primer sequences are listed in Supplementary Table S1.

### Immunoblots

Protein lysates were prepared as described earlier [4]. Lysates were separated on 8–12% SDS–polyacrylamide gels and electrically transferred onto PVDF membranes (Millipore, Billerica, MA, USA). Membranes were blocked for one hour at room temperature prior to anti-ROR1 (R&D Systems polyclonal Ab #AF2000) or anti-ROR2 (Sigma Prestige polyclonal Ab #HPA021868) antibody incubation at 4°C overnight. After washing with 0.01% Tris-buffered saline/Tween (TBS–Tween), membranes were incubated in secondary antibody for one hour. After further washing with TBS–Tween, membranes were developed using an enhanced chemiluminescence reagent and analysed using an ImageQuant LAS4000 (GE Healthcare Life Sciences).

### Proliferation assay

Approximately 6–8 hours after transfection, cells were trypsinised, counted, and seeded on to a 96 well plate in triplicates at a concentration of 1 × 10^4^ cells/mL for OVCAR3 and 2 × 10^4^ cells/mL for TOV112D. One column of media only was added for background reading. Cells were incubated and periodically analysed using the CCK-8 kit (Dojindo, Rockville USA) according to manufacturer's instructions. Readings were obtained 24, 48 and 72 hours after transfection. An increase in absorbance indicated an increase in cell number and significance was determined using the students paired *t* test.

### Adhesion assay

Tissue culture plates (Nunc) were coated with solutions of type I collagen (10 μg/ml) (Sigma-Aldrich), fibronectin (5 μg/ ml) (Millipore) or 3% bovine serum albumin (BSA) (SigmaAldrich) in phosphate-buffered saline (PBS) (Gibco, Carlsbad, CA, USA) and adhesion assays performed as described previously [10].

### Migration assay (wound healing)

Wound healing was analysed using IBIDI Culture-Inserts (IBIDI GmbH, Martinsried, Germany). Twenty-four hours after siRNA transfection, cells were dissociated from plates using 0.05% (w/v) trypsin and seeded into culture-insert plates at a concentration of 3 × 10^4^ cells per culture well for OVCAR3 and 5 × 10^4^ cells per culture well for TOV112D. After further 24 hours incubation, culture inserts were removed. Photographs of the movement of cells into the scratch area were taken every 6–12 hours until scratch area had closed using a Leica DMIL microscope (Leica Microsystems, North Ryde, NSW, AU). Wound healing was then analysed using TScratch software [34].

### Migration assay (transwell)

Cell migration was measured using Transwell inserts (Corning Life Sciences, Tewksbury, MA) according to manufacturer's instructions. Briefly, 48 hours following transfection, cells were counted and placed in transwell inserts at a concentration of 1 × 10^5^ cells per insert for OVCAR3 and 2 × 10^5^ cells per insert for TOV112D. For the OVCAR3 cells, the transwell inserts were coated approximately 12 hours prior to assay with type I collagen (10 μg/ml) (Sigma-Aldrich) to aid cell adhesion. After 24 hours incubation, cells were fixed with 100% methanol and stained with 1% crystal violet. The membrane was then removed and mounted on a glass slide. Images were taken of four areas of the membrane which were then analysed using ImageJ (Java Software). An average cell count of the four images was then used in statistical analysis, and all experiments were repeated in triplicate.

### Invasion assay

Cell invasion was measured using matrigel pre-coated transwell inserts (BioCoat Matrigel Invasion Chambers, Corning Life Sciences, Tewksbury, MA) according to manufacturer's instructions. Briefly, 24 hours following transfection, cells were counted and placed in transwell inserts (which were rehydrated with SFM for 2 hours prior) at a concentration of 1 × 10^5^ cells per insert for OVCAR3 and 4 × 10^5^ cells per insert for TOV112D. After 48 hours incubation, cells were fixed with 100% methanol and stained with 1% crystal violet. The membrane was then removed and mounted on a glass slide. Images were taken of four areas of the membrane which were then analysed using ImageJ (Java Software). An average cell count of the four images was then used in statistical analysis and all experiments were repeated in triplicate.

### Luciferase assay

A Wnt reporter luciferase assay was used to determine the effect of ROR2 and Wnt5a knockdown on β-catenin mediated TCF/LEF transcription. 24 hours after siRNA transfection, cells were trypsinised and plated in triplicate wells in a white bottomed 96 well plate at 6 × 10^5^ cells/ml. They were then left to adhere at 37°C whilst luciferase transfection mix was prepared. Cells were transfected with either 200 ng of TOP plasmid or 200 ng of FOP plasmid, in addition to 200ng of Renilla plasmid using 3 ul of Lipofectamine (Invitrogen, Carlsbad, CA, USA) per well. After 6–8 hours, transfection mix was removed and replaced with complete media. Triplicate wells were then stimulated with 40 ng/ul of Wnt3a (#5036-WN-010/CF R&D Systems, Minneapolis, USA) and Wnt5a (#645-WN-010, R&D Systems, Minneapolis, USA) alongside an un-stimulated control over night. The following day, firefly luciferase and renilla luminescence was read using the Dual-Glo Luciferase assay system (Promega) on the Glomax 96 Microplate Luminometer (Turner Biosystems Instrument, Sunnyvale CA, USA) according to the manufacturer's protocol. Relative β-catenin driven transcription activity was calculated as a TOP/FOP ratio.

### Statistical analysis

In order to compare ROR2 protein expression levels between benign and cancer, generalised linear mixed effects models were performed (GLMM). These kinds of models are suitable for repeated measure data. To simplify this analysis, intensities were grouped in < 1 and > = 1. Results are presented as *p*-values of the corresponding comparison.

In order to assess the association between ROR2 expression and clinicopathological parameters, Chi-square tests were calculated. These tests do not account for repeated measure data and are purely exploratory.

In order to examine time to event data (death, relapse) Cox-regressions were performed.

This model allows several entries for each subject, Details are described in [35]. Evaluations were done using R version 3.0.1.

All *in vitro* experimental results are expressed as mean ± standard deviation (SD). To correctly analyse statistical significance, an F-test was first used to determine equal or unequal data variance (standard deviation) with a value of < 0.05 determining unequal variance, and a value > 0.05 determining equal variance. If equal variance, a student's *t* test type 2 was used to determine significance. If unequal variance, a student's *t* test type 3 was used to determine significance. T test values below *P* < 0.05 were considered statistically significant. *P < 0.05, **P < 0.01, ***P < 0.001.

## SUPPLEMENTARY FIGURES AND TABLE



## References

[1] The Cancer Genome Atlas (2011). Integrated genomic analyses of ovarian carcinoma. Nature.

[2] Tothill RW, Tinker AV, George J, Brown R, Fox SB, Lade S, Johnson DS, Trivett MK, Etemadmoghadam D, Locandro B, Traficante N, Fereday S, Hung JA, Chiew YE, Haviv I, Australian Ovarian Cancer Study G (2008). Novel molecular subtypes of serous and endometrioid ovarian cancer linked to clinical outcome. Clin Cancer Res.

[3] Tan TZ, Miow QH, Huang RY, Wong MK, Ye J, Lau JA, Wu MC, Bin Abdul Hadi LH, Soong R, Choolani M, Davidson B, Nesland JM, Wang LZ, Matsumura N, Mandai M, Konishi I (2013). Functional genomics identifies five distinct molecular subtypes with clinical relevance and pathways for growth control in epithelial ovarian cancer. EMBO molecular medicine.

[4] Loh YN, Hedditch EL, Baker LA, Jary E, Ward RL, Ford CE (2013). The Wnt signalling pathway is upregulated in an *in vitro* model of acquired tamoxifen resistant breast cancer. BMC Cancer.

[5] Ford CE, Punnia-Moorthy G, Henry CE, Llamosas E, Nixdorf S, Olivier J, Caduff R, Ward RL, Heinzelmann-Schwarz V (2014). The non-canonical Wnt ligand, Wnt5a, is upregulated and associated with epithelial to mesenchymal transition in epithelial ovarian cancer. Gynecologic Oncology.

[6] Arend RC, Londono-Joshi AI, Straughn JM, Buchsbaum DJ (2013). The Wnt/beta-catenin pathway in ovarian cancer: a review. Gynecologic Oncology.

[7] Ford CE, Jary E, Ma SS, Nixdorf S, Heinzelmann-Schwarz VA, Ward RL (2013). The Wnt gatekeeper SFRP4 modulates EMT, cell migration and downstream Wnt signalling in serous ovarian cancer cells. PLoS ONE [Electronic Resource].

[8] Su HY, Lai HC, Lin YW, Liu CY, Chen CK, Chou YC, Lin SP, Lin WC, Lee HY, Yu MH (2010). Epigenetic silencing of SFRP5 is related to malignant phenotype and chemoresistance of ovarian cancer through Wnt signaling pathway. Int J Cancer.

[9] Ford CE, Qian Ma SS, Quadir A, Ward RL (2013). The dual role of the novel Wnt receptor tyrosine kinase, ROR2, in human carcinogenesis. International Journal of Cancer.

[10] Henry C, Quadir A, Hawkins NJ, Jary E, Llamosas E, Kumar D, Daniels B, Ward RL, Ford CE (2015). Expression of the novel Wnt receptor ROR2 is increased in breast cancer and may regulate both beta-catenin dependent and independent Wnt signalling. J Cancer Res Clin Oncol.

[11] Lara E, Calvanese V, Huidobro C, Fernandez AF, Moncada-Pazos A, Obaya AJ, Aguilera O, Gonzalez-Sancho JM, Sanchez L, Astudillo A, Munoz A, Lopez-Otin C, Esteller M, Fraga MF (2010). Epigenetic repression of ROR2 has a Wnt-mediated, pro-tumourigenic role in colon cancer. Mol Cancer.

[12] Rasmussen NR, Wright TM, Brooks SA, Hacker KE, Debebe Z, Sendor AB, Walker MP, Major MB, Green J, Wahl GM, Rathmell WK (2013). Receptor tyrosine kinase-like orphan receptor 2 (Ror2) expression creates a poised state of Wnt signaling in renal cancer. Journal of Biological Chemistry.

[13] Sun B, Ye X, Lin L, Shen M, Jiang T (2015). Up-regulation of ROR2 is associated with unfavorable prognosis and tumor progression in cervical cancer. International journal of clinical and experimental pathology.

[14] Huang J, Fan X, Wang X, Lu Y, Zhu H, Wang W, Zhang S, Wang Z (2015). High ROR2 expression in tumor cells and stroma is correlated with poor prognosis in pancreatic ductal adenocarcinoma. Scientific reports.

[15] Lu C, Wang X, Zhu H, Feng J, Ni S, Huang J (2015). Over-expression of ROR2 and Wnt5a cooperatively correlates with unfavorable prognosis in patients with non-small cell lung cancer. Oncotarget.

[16] Zhang S, Chen L, Cui B, Chuang HY, Yu J, Wang-Rodriguez J, Tang L, Chen G, Basak GW, Kipps TJ (2012). ROR1 is expressed in human breast cancer and associated with enhanced tumor-cell growth. PLoS One.

[17] Cui B, Zhang S, Chen L, Yu J, Widhopf GF, Fecteau JF, Rassenti LZ, Kipps TJ (2013). Targeting ROR1 inhibits epithelial-mesenchymal transition and metastasis. Cancer Res.

[18] Zhang H, Qiu J, Ye C, Yang D, Gao L, Su Y, Tang X, Xu N, Zhang D, Xiong L, Mao Y, Li F, Zhu J (2014). ROR1 expression correlated with poor clinical outcome in human ovarian cancer. Scientific reports.

[19] Zhang S, Cui B, Lai H, Liu G, Ghia EM, Widhopf GF, Zhang Z, Wu CC, Chen L, Wu R, Schwab R, Carson DA, Kipps TJ (2014). Ovarian cancer stem cells express ROR1, which can be targeted for anti-cancer-stem-cell therapy. Proc Natl Acad Sci U S A.

[20] Jacob F, Ukegijni K, Nixdorf S, Ford C, Olivier J, Caduff R, Scurry J, Guertler R, Hornung D, Mueller R, Fink D, Hacker N, Heinzelmann-Schwarz V (2012). Loss of secreted frizzled-related protein 4 correlates with an aggressive phenotype and predicts poor outcome in ovarian cancer patients. PLoS One.

[21] Rasmussen NR, Debebe Z, Wright TM, Brooks SA, Sendor AB, Brannon AR, Hakimi AA, Hsieh JJ, Choueiri TK, Tamboli P, Maranchie JK, Hinds P, Wallen EM, Simpson C, Norris JL, Janzen WP (2014). Expression of Ror2 mediates invasive phenotypes in renal cell carcinoma. PLoS One.

[22] Nomachi A, Nishita M, Inaba D, Enomoto M, Hamasaki M, Minami Y (2008). Receptor tyrosine kinase Ror2 mediates Wnt5a-induced polarized cell migration by activating c-Jun N-terminal kinase via actin-binding protein filamin A. J Biol Chem.

[23] He F, Xiong W, Yu X, Espinoza-Lewis R, Liu C, Gu S, Nishita M, Suzuki K, Yamada G, Minami Y, Chen Y (2008). Wnt5a regulates directional cell migration and cell proliferation via Ror2-mediated noncanonical pathway in mammalian palate development. Development.

[24] Nishita M, Itsukushima S, Nomachi A, Endo M, Wang Z, Inaba D, Qiao S, Takada S, Kikuchi A, Minami Y (2010). Ror2/Frizzled complex mediates Wnt5a-induced AP-1 activation by regulating Dishevelled polymerization. Mol Cell Biol.

[25] Fukuda T, Chen L, Endo T, Tang L, Lu D, Castro JE, Widhopf GF, Rassenti LZ, Cantwell MJ, Prussak CE, Carson DA, Kipps TJ (2008). Antisera induced by infusions of autologous Ad-CD154-leukemia B cells identify ROR1 as an oncofetal antigen and receptor for Wnt5a. Proc Natl Acad Sci U S A.

[26] Karin M (2009). NF-kappaB as a critical link between inflammation and cancer. Cold Spring Harbor perspectives in biology.

[27] Yamaguchi T, Yanagisawa K, Sugiyama R, Hosono Y, Shimada Y, Arima C, Kato S, Tomida S, Suzuki M, Osada H, Takahashi T (2012). NKX2–1/TITF1/TTF-1-Induced ROR1 is required to sustain EGFR survival signaling in lung adenocarcinoma. Cancer Cell.

[28] Tseng H-C, Kao H-W, Ho M-R, Chen Y-R, Lin T-W, Lyu P-C, Lin W-C (2011). Cytoskeleton Network and Cellular Migration Modulated by Nuclear-localized Receptor Tyrosine Kinase ROR1. Anticancer research.

[29] Tseng HC, Lyu PC, Lin WC (2010). Nuclear localization of orphan receptor protein kinase (Ror1) is mediated through the juxtamembrane domain. BMC Cell Biol.

[30] Choi MY, Widhopf GF, Wu CC, Cui B, Lao F, Sadarangani A, Cavagnaro J, Prussak C, Carson DA, Jamieson C, Kipps TJ (2015). Pre-clinical Specificity and Safety of UC-961, a First-In-Class Monoclonal Antibody Targeting ROR1. Clinical lymphoma, myeloma & leukemia.

[31] Hojjat-Farsangi M, Khan AS, Daneshmanesh AH, Moshfegh A, Sandin A, Mansouri L, Palma M, Lundin J, Osterborg A, Mellstedt H (2013). The tyrosine kinase receptor ROR1 is constitutively phosphorylated in chronic lymphocytic leukemia (CLL) cells. PLoS One.

[32] Jacob F, Guertler R, Naim S, Nixdorf S, Fedier A, Hacker NF, Heinzelmann-Schwarz V (2013). Careful selection of reference genes is required for reliable performance of RT-qPCR in human normal and cancer cell lines. PloS one.

[33] Vandesompele J, De Preter K, Pattyn F, Poppe B, Van Roy N, De Paepe A, Speleman F (2002). Accurate normalization of real-time quantitative RT-PCR data by geometric averaging of multiple internal control genes. Genome Biol.

[34] Geback T, Schulz MM, Koumoutsakos P, Detmar M (2009). TScratch: a novel and simple software tool for automated analysis of monolayer wound healing assays. BioTechniques.

[34] Therneau T, Grambsch P (2000). Modeling Survival Data: Extending the Cox Model.

