# Supplementary material for: Targeting the ROR1 and ROR2 receptors in epithelial ovarian cancer inhibits cell migration and invasion
Source: Oncotarget. 2015 Oct 20;6(37):40310–26. doi: 10.18632/oncotarget.5643 (PMC4741897; doi:10.18632/oncotarget.5643)
Supplement: Supplementary file 1 [file oncotarget-06-40310-s001.pdf]

SUPPLEMENTARY FIGURES AND TABLE

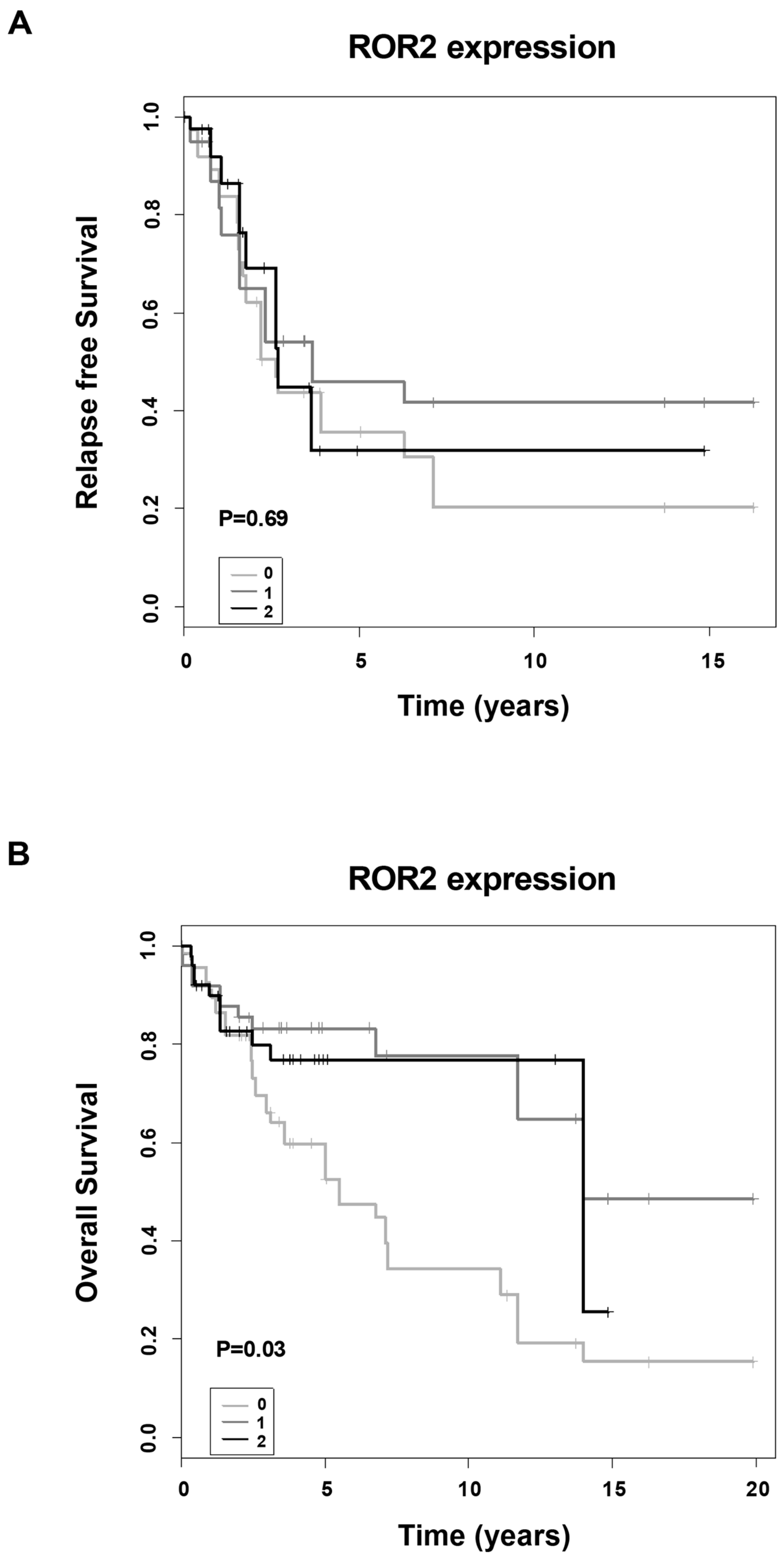

Supplementary Figure S1: ROR2 expression and patient survival. A. Kaplan-Meier Curve for relapse free survival as stratified by ROR2 expression, **B.** Kaplan-Meier Curve for overall free survival as stratified by ROR2 expression.

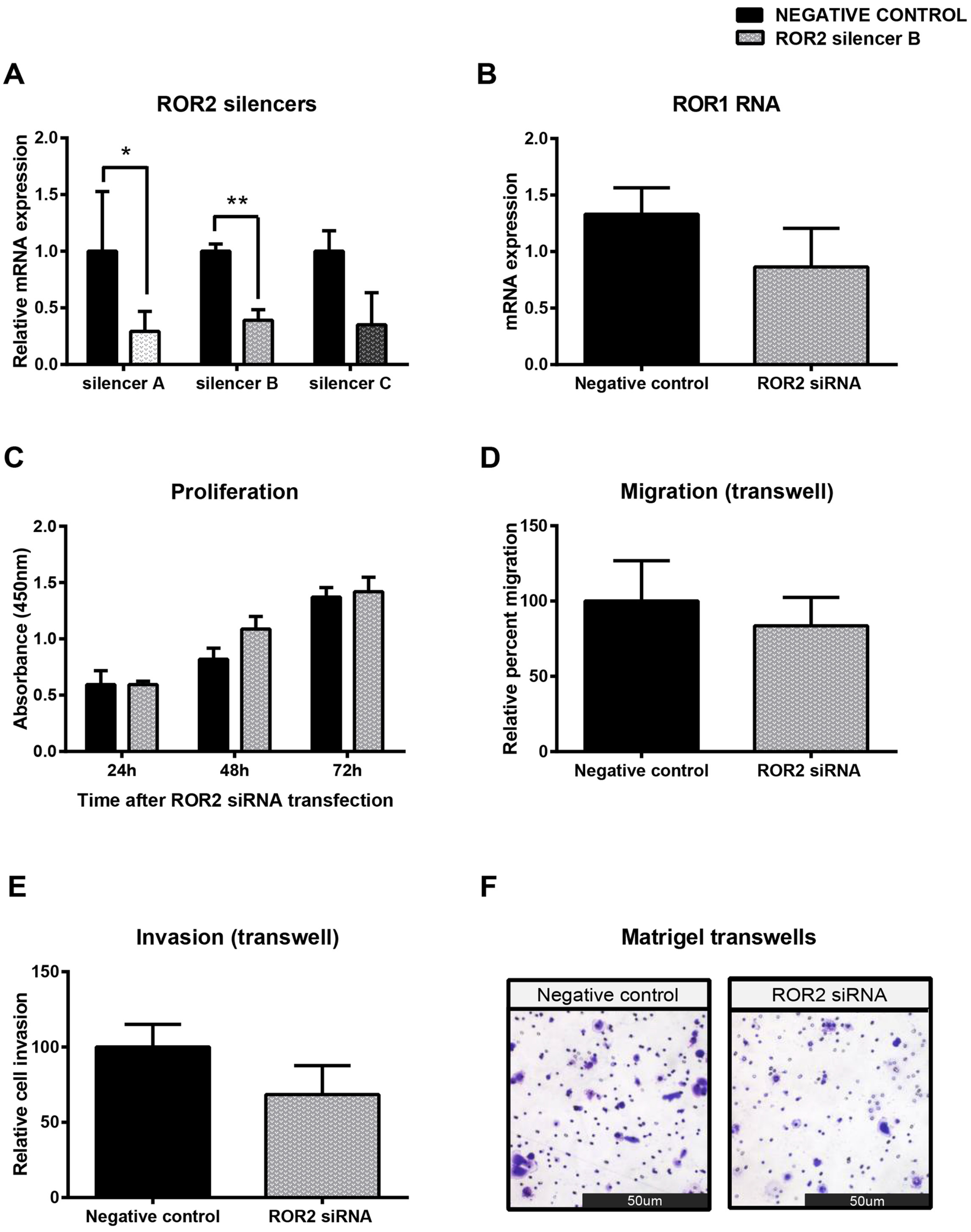

Supplementary Figure S2: Validation of ROR2 silencer. A. Two additional ROR2 silencers, B and C were compared to the originally used siRNA and showed similar knockdown levels. qRT-PCR was performed in triplicate and normalised to three different housekeeping genes (SDHA, HSPCB, RPL13A). Results represent an average of three experiments. Error bars represent the s.d of the mean. **B.** ROR2 knockdown using alternative silencer B had no effect on ROR1 mRNA levels. qRT-PCR was performed in triplicate and normalised to three different housekeeping genes (SDHA, HSPCB, RPL13A). Results represent an average of three experiments. Error bars represent the s.d of the mean. **C.** ROR2 knockdown using alternative silencer B had no effect on OVCAR3 proliferation. Results represent the average of three independent experiments. Error bars represent the s.d of the mean. **D.** Relative cell migration performed using the transwell migration assay is slightly decreased following ROR2 alternative siRNA knockdown in OVCAR3 cells. Results represent an average of three experiments. Error bars represent the s.d of the mean. **E.** Relative cell invasion performed using the matrigel pre coated transwell assay slightly decreased following ROR2 alternative siRNA knockdown in OVCAR3 cells. Results represent the average of three experiments. Error bars represent the s.d of the mean. **F.** Representative picture of OVCAR3 cells invading matrigel over 48 hours.

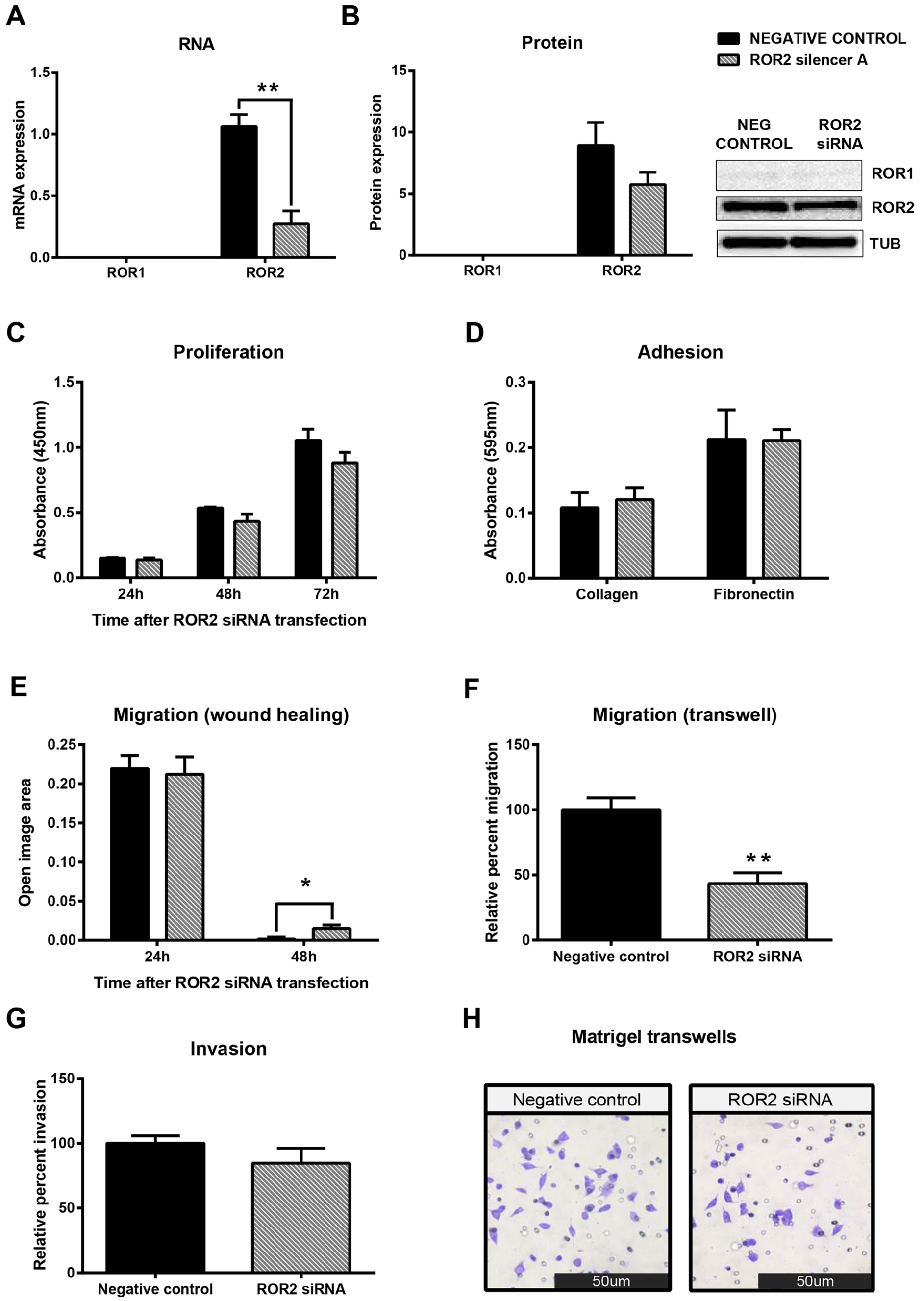

Supplementary Figure S3: ROR2 knockdown in the endometrioid cell line TOV112D. A. ROR2 is decreased at the mRNA level following siRNA (A) induced knockdown in TOV112D. No effect on ROR1 mRNA level. qRT-PCR was performed in triplicate and normalised to three different housekeeping genes (SDHA, HSPCB, RPL13A). Results represent an average of four experiments. Error bars represent the s.d of the mean. $**P < 0.01$. **B.** Densitometric analysis of ROR1 and ROR2 protein levels from three separate experiments. Representative immunoblots showing ROR2 knockdown at the protein level in TOV112D cells. No effect on ROR1 protein level. Top panel: ROR1, middle panel: ROR2, bottom panel: α-tubulin. **C.** Cell proliferation is slightly decreased following ROR2 knockdown in TOV112D cells over a 48–72 hours period, but not significantly. Results represent the average of three independent experiments. Error bars represent the s.d of the mean. **D.** ROR2 knockdown has no effect on the adhesion of TOV112D cells to collagen or fibronectin. Results represent the average of 3 experiments. **E.** Cell migration performed using the wound healing assay is significantly decreased following ROR2 knockdown in TOV112D cells. Results represent an average of three experiments. Error bars represent the s.d of the mean. $*P < 0.05$. **F.** Relative cell migration performed using the transwell migration assay is significantly decreased following ROR2 knockdown in TOV112D cells. Results represent an average of three experiments. Error bars represent the s.d of the mean. $**P < 0.01$. **G.** Relative cell invasion performed using the matrigel pre coated transwell assay slightly decreased following ROR2 knockdown in TOV112D cells. Results represent the average of three experiments. Error bars represent the s.d of the mean. **H.** Representative picture of TOV112D cells invading matrigel for 48 hours period.

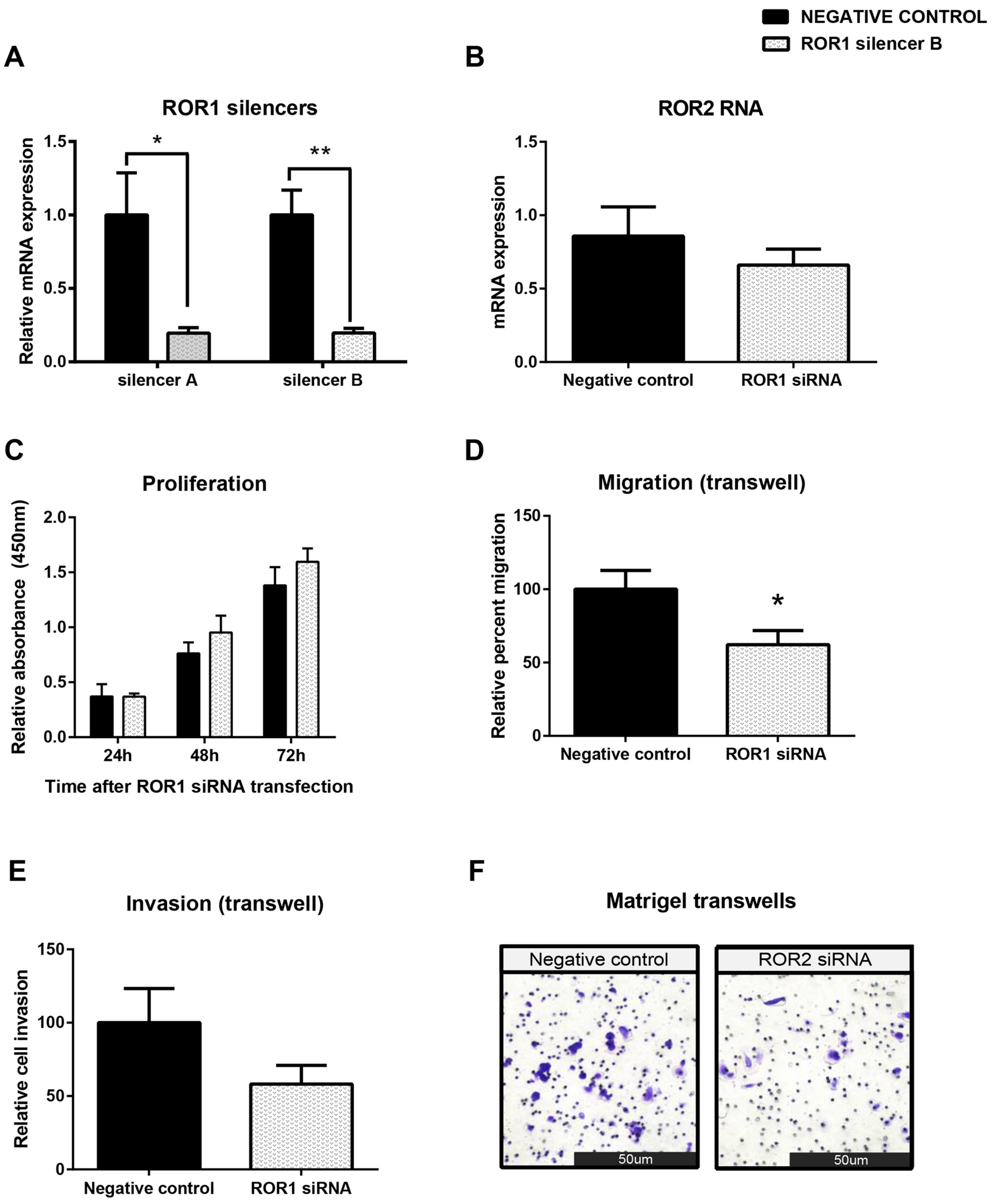

Supplementary Figure S4: Validation of ROR1 silencer. A. One additional ROR1 silencer was compared to the originally used siRNA and showed similar knockdown levels. qRT-PCR was performed in triplicate and normalised to three different housekeeping genes (SDHA, HSPCB, RPL13A). Results represent an average of three experiments. Error bars represent the s.d of the mean. **B.** ROR1 knockdown using alternative silencer B had no effect on ROR2 mRNA levels. qRT-PCR was performed in triplicate and normalised to three different housekeeping genes (SDHA, HSPCB, RPL13A). Results represent an average of three experiments. Error bars represent the s.d of the mean. **C.** ROR1 knockdown using alternative silencer B had no effect on OVCAR3 proliferation. Results represent the average of three independent experiments. Error bars represent the s.d of the mean. **D.** Relative cell migration performed using the transwell migration assay is significantly decreased following ROR1 alternative siRNA B knockdown in OVCAR3 cells. Results represent an average of three experiments. Error bars represent the s.d of the mean. $^*P < 0.05$. **E.** Relative cell invasion performed using the matrigel pre coated transwell assay slightly decreased following ROR1 alternative siRNA knockdown in OVCAR3 cells. Results represent the average of three experiments. Error bars represent the s.d of the mean. $P = 0.0525$. **F.** Representative picture of OVCAR3 cells invading matrigel over 48 hours.

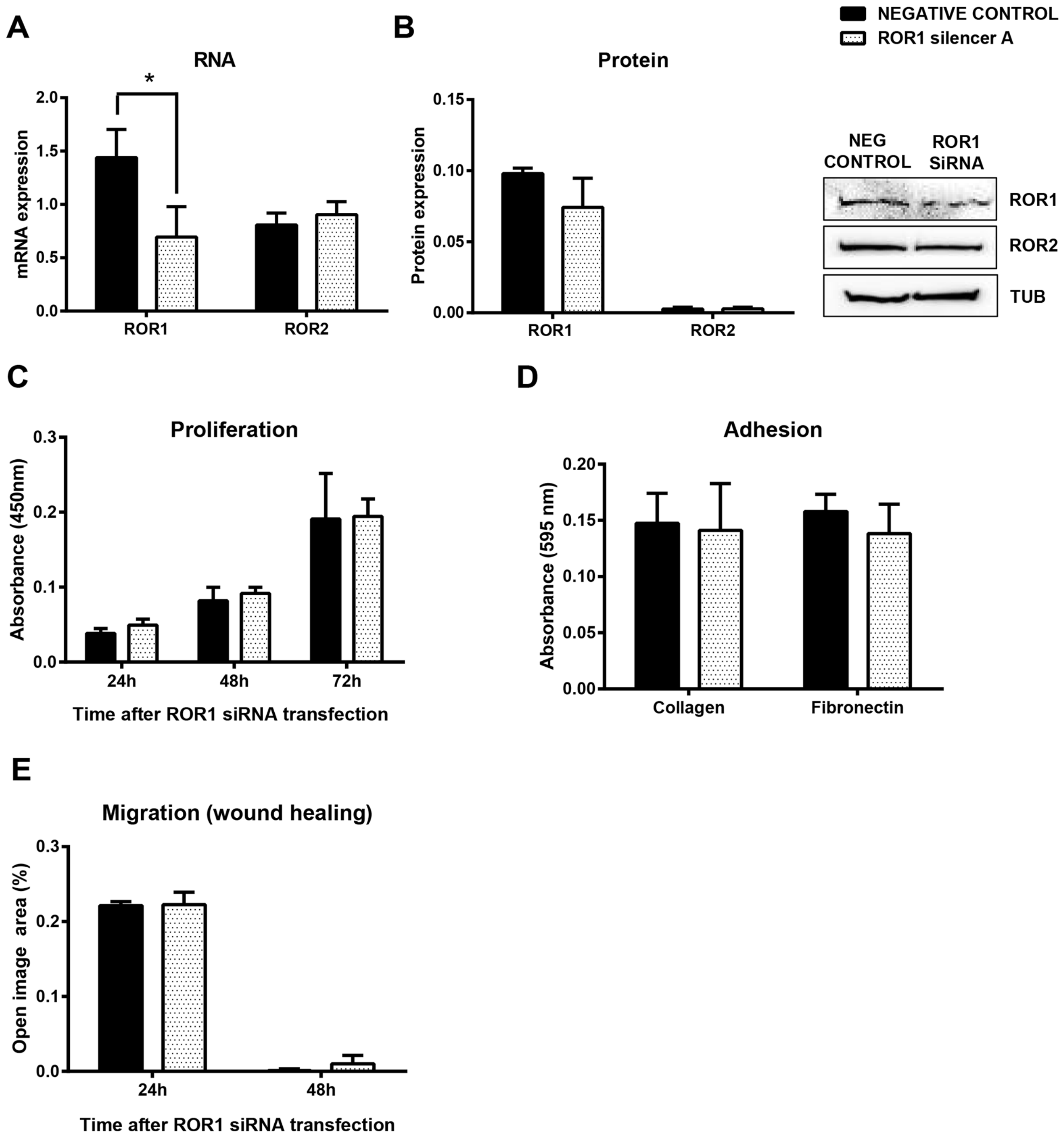

Supplementary Figure S5: Knockdown of ROR1 in normal human ovarian surface epithelial cells has no effect. A. ROR1 is decreased at the mRNA level following siRNA (A) induced knockdown in normal human ovarian surface epithelial (HOSE6.3) cells. No effect on ROR2 mRNA level. qRT-PCR was performed in triplicate and normalised to three different housekeeping genes (SDHA, HSPCB, RPL13A). Results represent an average of three experiments. Error bars represent the s.d of the mean. $*P < 0.05$. **B.** Densitometric analysis of ROR1 and ROR2 protein levels from three separate experiments. Representative immunoblots showing ROR1 knockdown at the protein level in HOSE6.3 cells. No effect on ROR2 protein level. Top panel: ROR1, middle panel: ROR2, bottom panel: α-tubulin. **C.** Cell proliferation does not change following ROR1 knockdown in HOSE6.3 cells over a 48–72 hour period. Results represent the average of three independent experiments. Error bars represent the s.d of the mean. **D.** ROR1 knockdown has no effect on the adhesion of HOSE6.3 cells to collagen or fibronectin. Results represent the average of 3 experiments and error bars represent the s.d of the mean. **E.** Cell migration performed using the wound healing assay does not change following ROR2 knockdown in HOSE6.3 cells. Results represent an average of three experiments. Error bars represent the s.d of the mean.

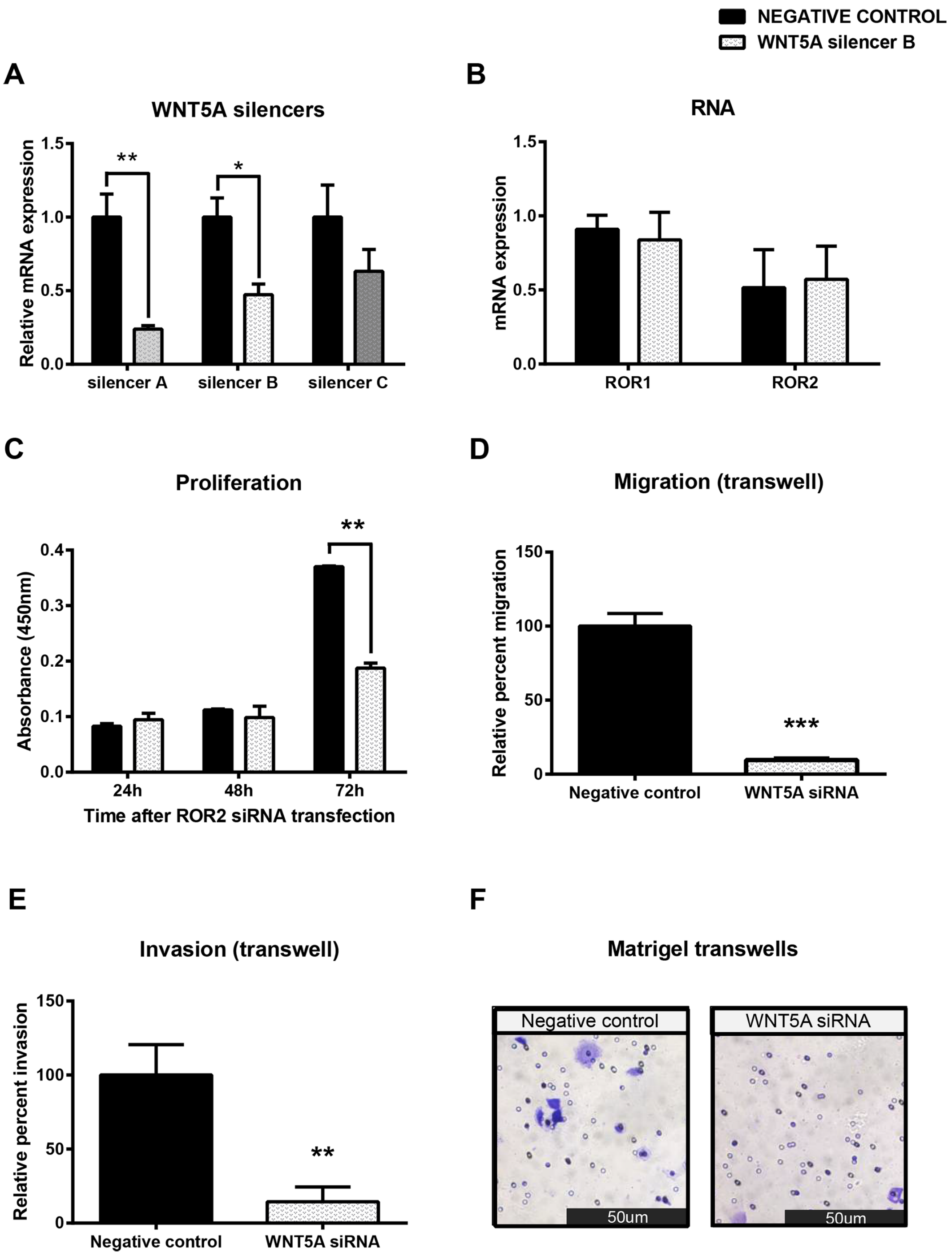

Supplementary Figure S6: Validation of WNT5A silencer. A. Two additional WNT5A silencers, B and C were compared to the originally used siRNA A and showed similar knockdown levels. qRT-PCR was performed in triplicate and normalised to three different housekeeping genes (SDHA, HSPCB, RPL13A). Results represent an average of three experiments. Error bars represent the s.d of the mean. **B.** WNT5A knockdown using alternative silencer B had no effect on ROR1 or ROR2 mRNA levels. qRT-PCR was performed in triplicate and normalised to three different housekeeping genes (SDHA, HSPCB, RPL13A). Results represent an average of three experiments. Error bars represent the s.d of the mean. **C.** WNT5A knockdown using alternative silencer B significantly decreased OVCAR3 proliferation. Results represent the average of three independent experiments. Error bars represent the s.d of the mean. $^{**}P < 0.01$. **D.** Relative cell migration performed using the transwell migration assay is significantly decreased following WNT5A alternative siRNA B knockdown in OVCAR3 cells. Results represent an average of three experiments. Error bars represent the s.d of the mean. $^{***}P < 0.01$. **E.** Relative cell invasion performed using the matrigel pre coated transwell assay significantly decreased following WNT5A alternative siRNA B knockdown in OVCAR3 cells. Results represent the average of three experiments. Error bars represent the s.d of the mean. $^{**}P < 0.01$. **F.** Representative picture of OVCAR3 cells invading matrigel over 48 hours.

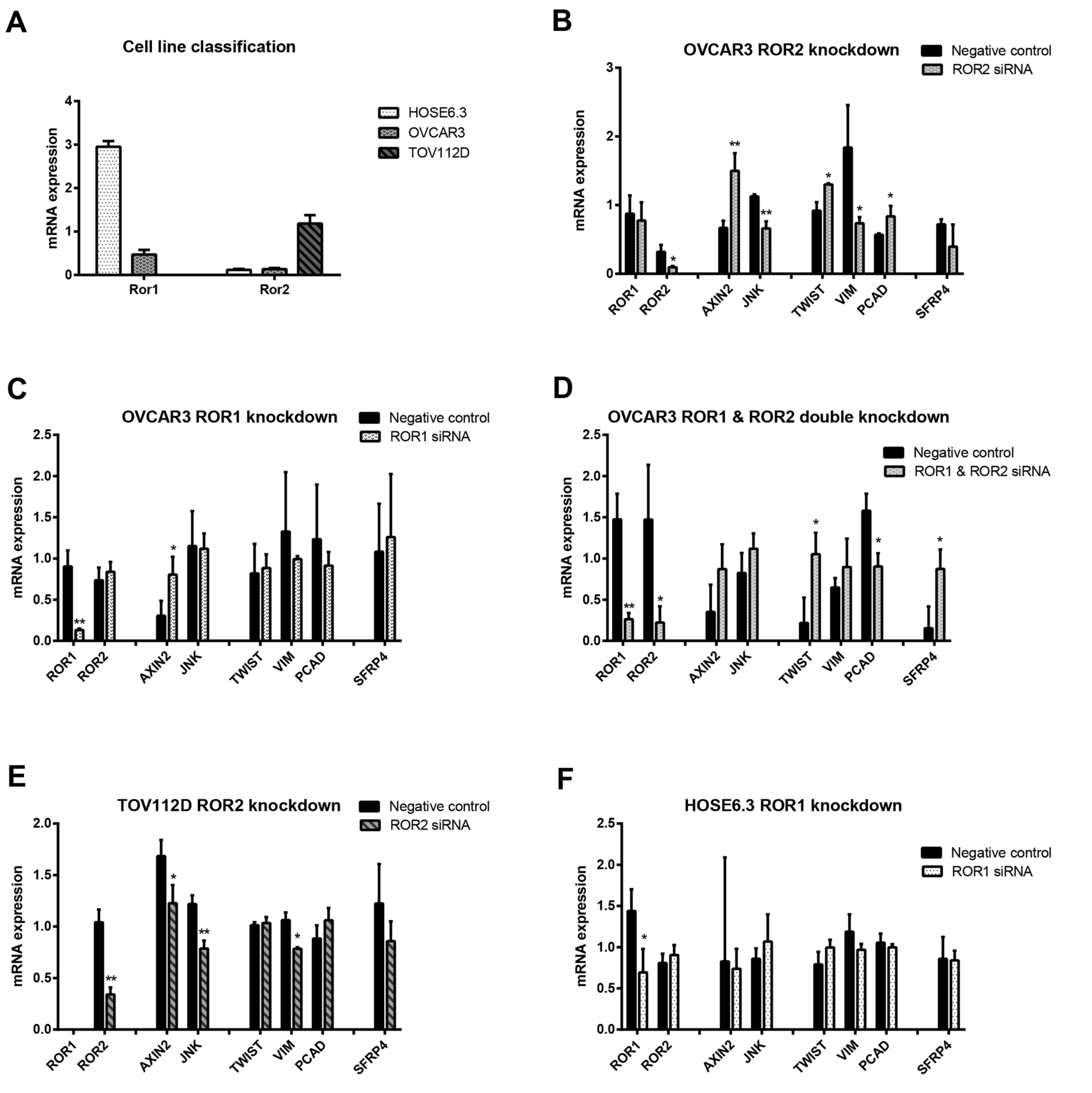

Supplementary Figure S7: Cell line classification and transcriptional profiles. qRT-PCR was performed in triplicate and normalised to three different housekeeping genes (SDHA, HSPCB, RPL13A). Results represent an average of three experiments. Error bars represent the s.d of the mean. $^{*}P < 0.05$, $^{**}P < 0.01$, $^{**}P < 0.001$. **A.** Expression of ROR1 and ROR2 mRNA in HOSE6.3, OVCAR3 and TOV112D cell lines. **B.** ROR2 knockdown in OVCAR3 significantly increases AXIN2, TWIST and PCADHERIN. **C.** ROR1 knockdown in OVCAR3 significantly increases AXIN2 but has no effect on JNK, TWIST, VIMENTI, PCADHERIN or SFRP4. **D.** Double ROR1 and ROR2 knockdown in OVCAR3 cells significantly increases TWIST, PCADHERIN and SFRP4 but has no effect on AXIN2, JNK and VIMENTIN. **E.** ROR2 Knockdown in TOV112D significantly decreases AXIN2, JNK and VIMENTIN but has no effect on TWIST, PCADHERIN or SFRP4. **F.** ROR1 knockdown in HOSE6.3 has no effect on WNT or EMT genes.

Supplementary Table S1: Primer sequences used for qRT-PCR

Gene	Forward	Reverse
ROR1	CAACAAGAAGCCTCCCTAATGG	CCTGAGTGACGGCACCTAGAA
ROR2	CGACTGCGAATCCAGGACC	CCGGTTGCCAATGAAGCGTG
WNT5A	CAGGCTTAACCCGGTCGC	CTGGCATTCTTTGATGCCTG
AXIN2	CTCAATTCGCGGGAGGGGGC	GAGCGGGAGCGGGGGCTATA
JNK	TCTGGTATGATCCTTCTGAAGCA	TCCTCCAAGTCCATAACTTCCTT
TWIST	GCCAATCAGCCACTGAAAGG	TGTTCTTATAGTTCCTCTGATTGTTACCA
VIM	CCAAACTTTTCCTCCCTGAACC	GTGATGCTGAGAAGTTTCGTTGA
CDH3	CACGACCTCATGTTCACCAT	CTCAGGGACTTTTTCCCGGT
SFRP4	TGTGTTACGAGTGGCG	GGGGGATTACTACGACTG
RPL13A	CCTGGAGGAGAAGAGGAAAGAGA	TTGAGGACCTCTGTGTATTTGTCAA
HSPCB	TCTGGGTATCGGAAAGCAAGCC	GTGCACTTCCTCAGGCATCTTG
SDHA	TGGGAACAAGAGGGCATCTG	CCACCACTGCATCAAATTCATG
